# Anisotropic Magnetoresistance Evaluation of Electrodeposited Ni_80_Fe_20_ Thin Film on Silicon

**DOI:** 10.3390/mi13111804

**Published:** 2022-10-22

**Authors:** Payam Khosravi, Seyyed Ali Seyyed Ebrahimi, Zahra Lalegani, Bejan Hamawandi

**Affiliations:** 1 Advanced Magnetic Materials Research Center, School of Metallurgy and Materials, College of Engineering, University of Tehran, Tehran 111554563, Iran; 2 Department of Applied Physics, KTH Royal Institute of Technology, SE-106 91 Stockholm, Sweden

**Keywords:** electrodeposition, permalloy, magnetoresistance, MOKE, AMR, FMR

## Abstract

In this study, a simple growth of permalloy NiFe (Py) thin films on a semiconductive Si substrate using the electrochemical deposition method is presented. The electrodeposition was performed by applying a direct current of 2 mA/cm^2^ during different times of 120 and 150 s and thin films with different thicknesses of 56 and 70 nm were obtained, respectively. The effect of Py thickness on the magnetic properties of thin films was investigated. Field emission scanning electron microscopy (FESEM), energy-dispersive X-ray spectroscopy (EDS), atomic force microscopy (AFM), ferromagnetic resonance (FMR), anisotropic magnetoresistance (AMR), and magneto-optic Kerr effect (MOKE) analyses were performed to characterize the Py thin films. It was observed that the coercivity of the Py thin film increases by increasing the thickness of the layer. Microscopic images of the layers indicated granular growth of the Py thin films with different roughness values leading to different magnetic properties. The magnetic resonance of the Py thin films was measured to fully describe the magnetic properties of the layers. The magnetoresistance ratios of deposited Py thin films at times of 120 and 150 s were obtained as 0.226% and 0.235%, respectively. Additionally, the damping constant for the deposited sample for 120 s was estimated as 1.36 × 10^−2^, which is comparable to expensive sputtered layers’ characteristics.

## 1. Introduction

Py thin films have played a very important role in the field of magnetism and spintronics due to their low magnetic anisotropy [1,2,3,4,5,6,7]. They have been used extensively in magneto-resistive devices with high efficiency [8,9,10]. Additionally, due to their very low damping parameter, they have been widely used for high-frequency spintronic devices which have a function based on the ferromagnetic resonance effect [11,12]. As electronic devices are still very dependent on semiconductor-based technology, coating Py on the surface of Si is of vital importance for magnetic device developments.

Today, physical methods such as molecular beam epitaxy [13], the ion-beam method [14], the vacuum evaporation technique [15], and sputtering [16] are commonly used for the fabrication of thin films. The advantages of these methods are obtaining pure materials, sharp interfaces, and high control of film growth but they require high vacuum so these techniques are complicated and expensive. However, electrodeposition is a fabrication technique that does not require a vacuum and is a relatively simple and inexpensive method [17,18]. To obtain high-quality deposits, the electrodeposition technique is a proper method to control the thickness, composition, and stoichiometry of alloys [19,20,21]. However, the deposition of thin films by electrodeposition requires special care. For example, contamination and fluctuations of the composition have an undesirable effect on the magnetic properties of the manufactured film [22].

There are many previous studies that have reported electrodeposited Py films onto metal substrates [23,24,25,26,27]. However, reports on the electrodeposition of Py on Si are not reported often, especially regarding magnetic characteristic investigations [28,29,30]. Silicon is an appropriate case as a substrate because it conducts properly well to allow electrodeposition without the need for a seed layer, leading to the fabricating of thin magnetic films matched with silicon technology [31]. It is significant to optimize the electrochemical conditions for Py electrodeposition onto semiconductor substrates to get the desired microstructure. In order to obtain the optimal thickness of Py for better adhesion between Py and substrate, Py thin films of submicrometer thickness were electrodeposited onto Si (111) surfaces by Gao et al. [32]. They reported that Py films were able to have proper adhesion to the Si substrate when the thickness of the initial deposited Py was more than 20 nm. They also determined the coercivity of samples with 150 nm thickness to be about 6.8 Oe. The skewed shape of the hysteresis of the magnetization curve for a thin film of Fe_x_Ni_1-x_ electrodeposited on Si (100) was presented by Spada et al. [33]. Additionally, it is found that using surfactants in the electrodeposition electrolyte can enhance the quality and adhesion. For example, strong adhesion of Py on Si substrate by electrodeposition from a solution containing saccharin was observed by Sam et al. [34].

In the present research, Py was electrodeposited directly onto a Si substrate without a seed layer. Despite not using organic additives such as saccharin in the electrodeposition solution, it was found that electroplating thin films represented reasonably strong adhesion to the Si substrates. The properly prepared Py thin films on the Si have the capacity to be used in technological elements. In particular, in this work, the static and dynamic magnetic properties of the electrodeposited Py thin films on the Si substrate were investigated. The main purpose of investigating the magnetic properties of the electrodeposited Py layers was to observe the anisotropic magnetoresistance (AMR) effect. The AMR signal was successfully detected. For the first time, a relatively comprehensive study of the magnetic properties of such a magnetic thin film via magneto transport, magneto-optic, and magneto resonance measurements were presented.

## 2. Experimental

### 2.1. Materials and Instrumentation

N-type silicon wafers (D.M.S Co., Hwaseong, South Korea), nickel (II) sulfate (NiSO_4_.6H_2_O, Merck, Darmstadt, Germany), iron (II) sulfate (FeSO_4_.7H_2_O, Merck, Germany), and boric acid (H_3_BO_3_, Merck, Germany) were used as primary materials to make the solution for electrodeposition.

Atomic force microscopy (AFM, NT-MDT TS150 ENTEGRA) was used to investigate the surface topography of the samples. The magneto-optic characteristic of prepared samples was examined by the magneto-optic Kerr effect (MOKE) based on differential intensity using a He-Ne laser. The light passed through a polarizer, then reflected by the sample and passed through an analyzer, and was monitored by a detector. Optical lenses were used to focus and regulate the laser beam before and after reflection, respectively. The magnetic field was applied parallel to the plane of incident laser light. Ferromagnetic resonance (FMR) was analyzed using a subminiature version A (SMA) connector. For this purpose, a radiofrequency (RF) field with a fixed output power of 16 dBm was applied to a 50 Ω micro-stripe line (200 µm width). The microwave frequency range of the external DC magnetic field was up to 20 GHz (step of 1 GHz). Anisotropic magnetoresistance (AMR) measurement was performed using a four-point probe setup (Keithley 2450). The morphology of the samples was studied by field emission scanning electron microscopy (FESEM, MIRA3TESCAN-XMU). Additionally, energy-dispersive spectroscopy (EDS) was used to determine the film composition.

### 2.2. Sample Preparation

#### 2.2.1. Solution

NiSO_4_.6H_2_O (0.4 M) was added to deionized water under stirring. After nickel sulfate was dissolved in deionized water at room temperature, FeSO_4_.7H_2_O (0.004 M) was added and stirred until dissolved. Then, H_3_BO_3_ (0.4 M) was added to the solution and the solution remained under stirring until it was homogenized.

It should be noted that before each use of the solution, it was placed in an ultrasound for 10 min to ensure its homogeneity.

#### 2.2.2. Electrochemical Deposition

The silicon wafer was cut out into 1.5 cm × 1.5 cm slices. In order to remove surface contamination such as surface oxide, the Si substrate was immersed in hydrofluoric acid (HF, 10% *v*/*v*) solution for 30 s, then washed with ethanol, acetone, and distilled water, respectively. After washing, the Si samples were instantly transferred to the electrodeposition cell to prevent oxidation of the silicon surface. A two-electrode cell system was utilized for the deposition of the Py layers with platinum as anode and Si substrate as a cathode. Two samples were electrodeposited at room temperature: the samples (S1 and S2) were electrodeposited by applying a direct current of 2 mA/cm^2^ for 120 s and 150 s, respectively.

## 3. Result and Discussion

**Figure 1:**
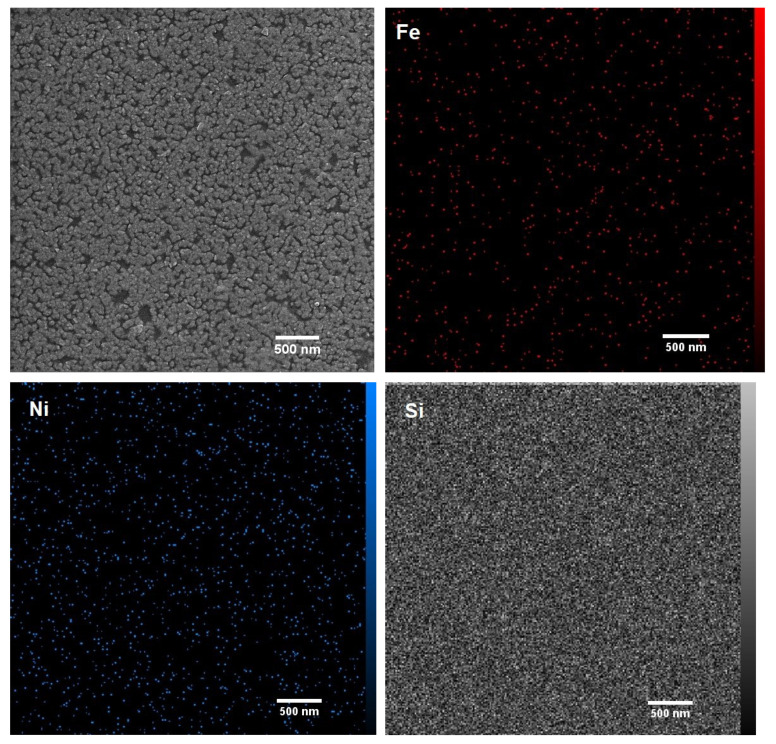
Morphology and element distribution map of sample S1.

Figure 1 shows the FESEM images and element distribution (EDS) map analysis of sample S1. As can be seen in Figure 1, the surface of the sample shows the island growth of Py grains. Additionally, according to Figure 1, the distribution of Ni and Fe in the layer is uniform, which indicates the homogeneity of the layer and confirms the success of the coating using the electrochemical method. It should be noted that it is very difficult to obtain a homogeneous layer in the process of electrochemical deposition because different parameters such as deposition time, applied voltage, current, and substrate can affect the process and each of them can cause non-uniformity in the deposited layer. In this study, the appropriate parameters for the deposition were determined after much trial and error to obtain a uniform layer.

**Figure 2:**
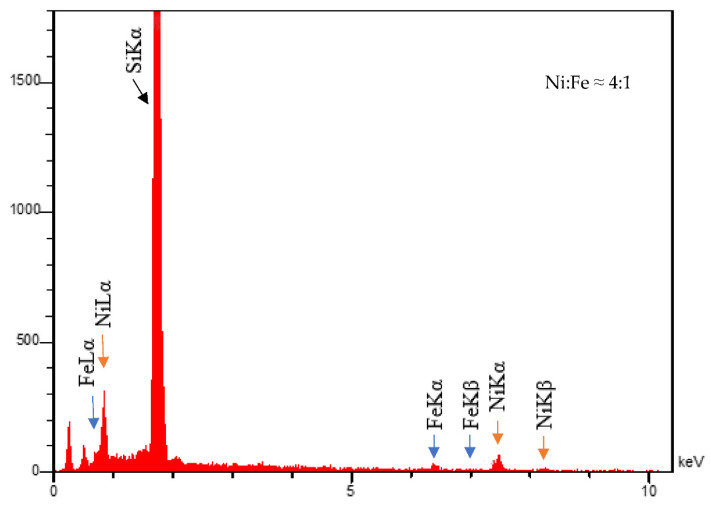
EDS analysis of sample S1.

Figure 2 shows the EDS analysis of sample S1. According to the EDS measurements, the Ni and Fe content of the layers were calculated and this shows that the ratio of the Ni to Fe is about 4:1, close to the composition of Ni_80_Fe_20_.

**Figure 3:**
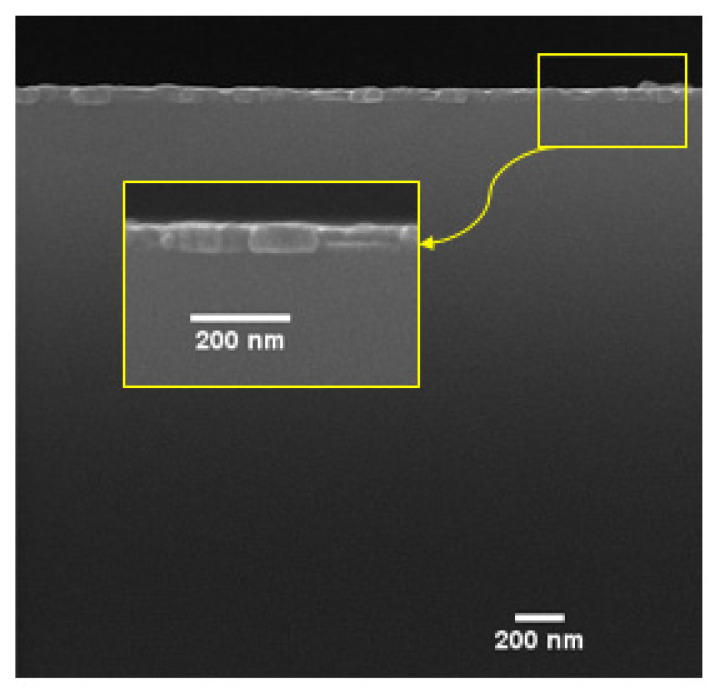
Cross-sectional FESEM image of sample S1.

The cross-sectional FESEM image of sample S1 is shown in Figure 3. According to Figure 3, the Py layer has an average thickness of about 56 nm, and it appears compact and fine in the structure. To estimate the thickness of the Py layer in sample S2, Faraday’s law can be used [35]. According to Faraday’s law, the thickness of coated layer with the electrochemical method can be determined by the following equation:

(1) $$T=\frac{\alpha \;i\;M\;t}{10^{-7}\;F\;S\;n\;\rho }$$

where *T* is the thickness of the layer, *t* is time, *i* is the operating electric current, *M* is the molar mass of the substance, *F* is Faraday’s constant, *S* is the area of electrodeposition, *n* is the number of electrons involved in an electrode reaction, ρ is the density of the substance, and α is the return coefficient which is equal to 1.516 here. The thickness of the S2 layer is estimated to be 70 nm by using Equation (1).

**Figure 4:**
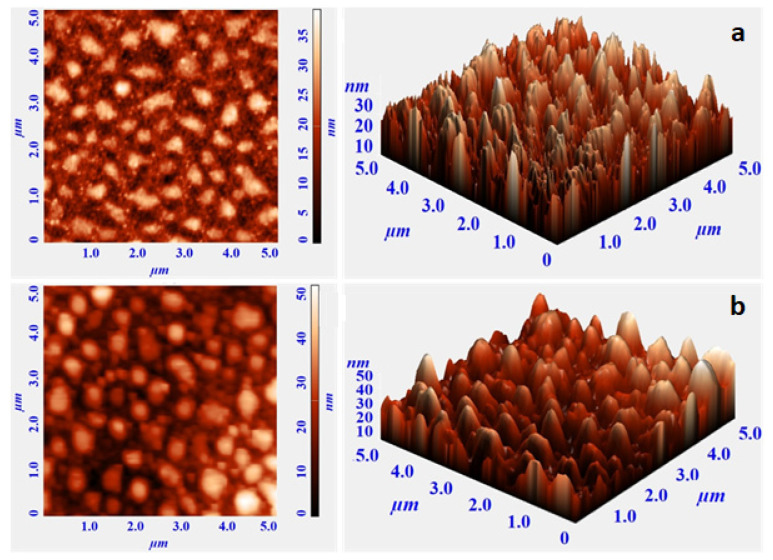
The 2D and 3D topographical images of (**a**) sample S1 and (**b**) sample S2.

AFM images analysis to investigate the surface morphologies (2D and 3D) of samples S1 and S2 are shown in Figure 4. AFM is a powerful technique to study the surface morphology at the nano- to micro-scale [36,37,38,39]. Topographical images from both samples were recorded over 5 µm × 5 µm scan areas. According to Figure 4, the grain size of both samples is in the nanometer range. It also shows that a rough surface was obtained by the electrodeposition method. According to the AFM results, a higher mean surface roughness (30 nm) is obtained for sample S2 deposited on Si in comparison with the mean surface roughness of sample S1 (22 nm). The AFM image of sample S1 shows regions with a typical rough shape and almost uniform grains. There are many sharp vertically aligned regions appearing in the topographical images of both samples S1 and S2. This vertical alignment can be the result of granular growth during the electroplating of the thin film. Electrodeposited samples in general give granular structure because the growth mode for electrochemical deposition of a metal (*M*) onto a substrate (*S*) follows the overall reaction [40]:

(2) $$M_{solution}^{+}+e^{-}\overset{Substrate}{\to }M_{lattice}$$

Determinant parameters in this reaction are the interaction energy between the metal adatoms and the substrate$\;\left(E_{M-S}\right)$, and the interaction energy or binding energy between the metal adatoms and the native substrate$\;\left(E_{M-N}\right)$. In electrodeposition, $E_{M-S}<E_{M-N}$ and there is a driving force for the depositing material to segregate on the surface and deposition occurs according to the Volmer–Weber island growth mechanism [41,42].

The magnetic properties of the prepared samples are measured through MOKE, magnetoresistance (MR), and FMR measurements. According to the MOKE analysis, by increasing the Py thickness, coercivity is increased from 1.3 Oe for S1 to 3.9 Oe for S2. This could be due to alteration of thickness and also surface roughness. Some reports have discussed the increase of coercivity by increasing the thickness and attributed it to the existence of the out-of-plane anisotropy component at the thicker samples [43,44,45,46]. MOKE analysis showed that with an increase in Kerr intensity the saturation magnetization (M_s_) is changed. Additionally, an increase in the thickness results in an increase in the interaction of light with the matter [47,48], hence both M_s_ and light–matter interaction are important.

**Figure 5:**
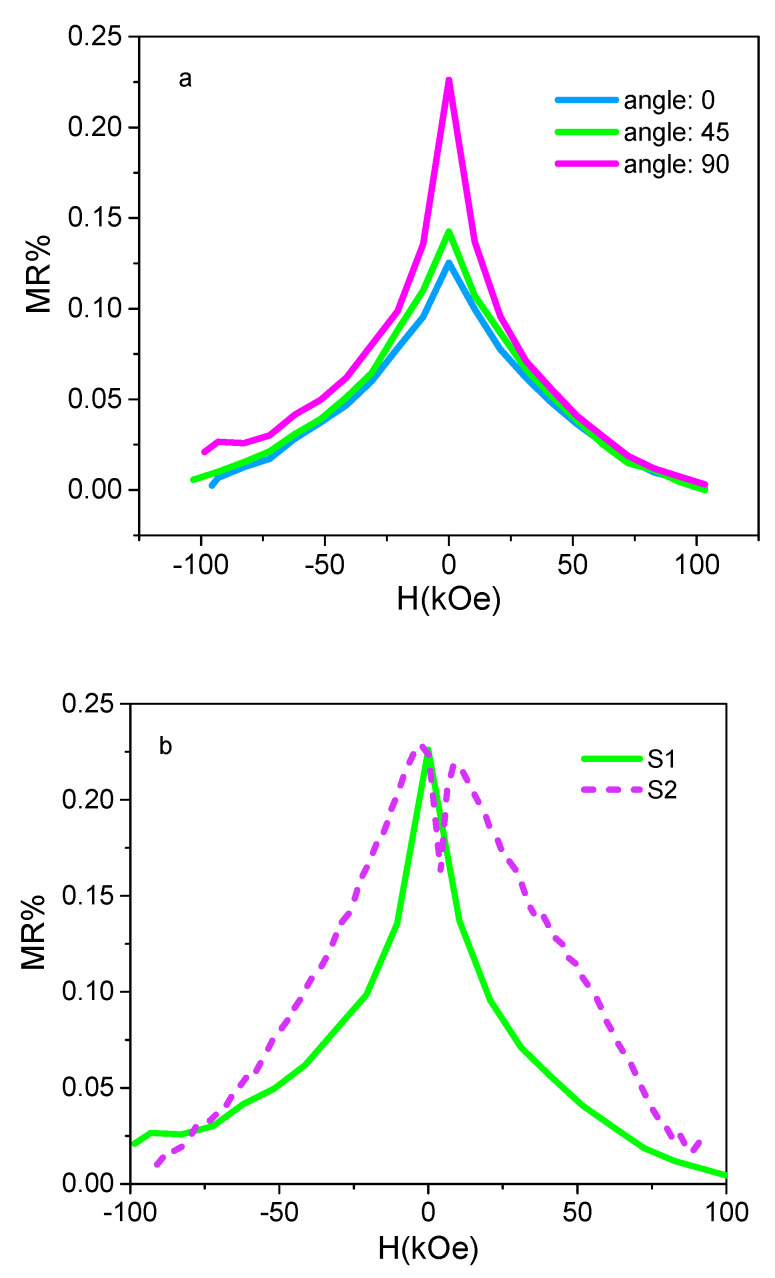
(**a**) AMR measurements for the S1 sample at different angles 0, 45, and $90^{o}$ between H and current; (**b**) AMR measurements for S1 and S2 samples at the angle $90^{o}$.

The MR property was measured with two probe modes at room temperature by measuring the resistance of the samples as a function of the external magnetic field (*H*). The MR ratio was defined as [49]:

(3) $$MR_{\left(H\right)}=\frac{\Delta \rho }{\rho_{0}}=\frac{\Delta R_{\left(H\right)}}{R_{0}}=\frac{R-R_{0}}{R_{0}}$$

where Δρ is the change in the sample resistivity, Δ*R_(H)_* is the change of the sample resistance due to the magnetic applied field, $\rho_{0}$ is the zero-field resistivity, *R*_0_ is the resistance of the sample, and *R* is the resistance in an external magnetic field *H*. Figure 5a shows the dependence of magnetoresistance and the angle between the electric field and the magnetic field. According to Figure 5a, for sample S1 the magnetoresistance increases by changing the angle between the electric field and magnetic field from 0 to 90. It can be due to the change in the angle between the magnetic easy-axis direction and the magnetic field [50]. According to Figure 5b, the MR ratios at the angle of 90 degrees are 0.226% and 0.235% for samples S1 and S2, respectively. According to previous studies [51,52], the magnitude of MR increases with the increase in thickness. AFM and FESEM studies showed that the thickness and roughness are larger in the case of sample S2 as compared to S1. A greater MR value in S2 compared to S1 could be due to the rough surface of S2. However, the effect of roughness and thickness has led to a negligible difference in the MR ratio. In comparison to other MR measurements of sputtered Py thin films, the MR ratios in this study are lower [53,54].

**Figure 6:**
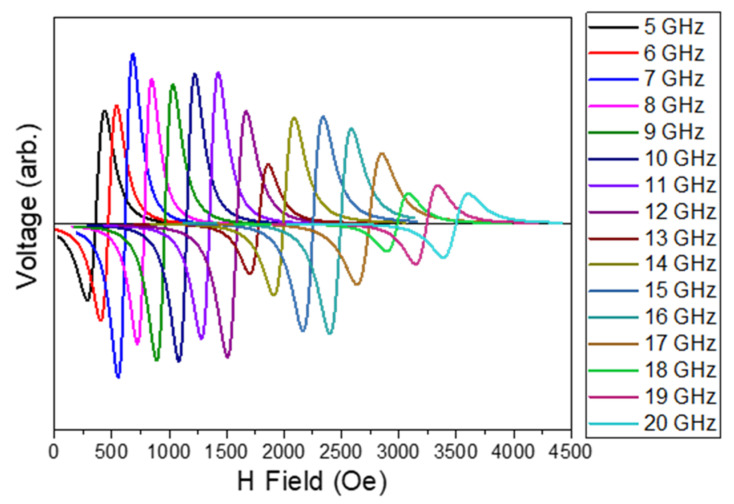
FMR analysis for sample S1.

FMR is a very powerful experimental technique in the study of ferromagnetic thin films. In the process of resonance, the energy is absorbed from the transverse magnetic field, which occurred when the frequency matched the Larmor frequency. The Larmor frequency depends on the orientation of the material and the strength of the magnetic field. The dependence of FMR frequency on the external magnetic field for thin films can be described by the Kittel formula [55]:

(4) $$f_{r}=\frac{\mu_{0}\gamma }{2\pi }\sqrt{\left(H+H_{k}+M_{s}\right)\left(H+H_{k}\right)}$$

where $\mu_{0}$ is the permeability of the free space, γ is the gyromagnetic ratio, *H* is the external magnetic field, $M_{s}$ is the saturation magnetization, and $H_{k}$ is the uniaxial anisotropy field, which is negligible for Py films with a thickness of 56 nm. FMR analysis was performed on sample S1 (with a thickness of 56 nm). The response of the sample to the FMR test is shown in Figure 6.

**Figure 7:**
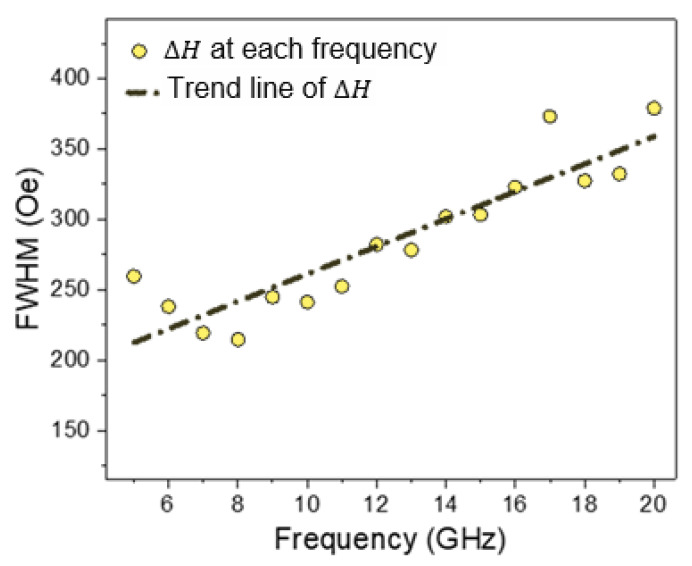
FMR analysis for sample S1 (FWHM vs. frequency).

Figure 7 shows the full width at half maximum (FWHM) of the resonance field peaks (ΔH) at each frequency. The damping constant can be derived from the FWHM [56]. According to Figure 7, the damping parameter was estimated as 1.36 × 10^−2^. Several studies reported the value of the damping constant to be about 0.6 × 10^−2^ using the sputtering method for the deposition of thin films [57,58,59]. Additionally, some studies reported different damping constants for the deposition of permalloy onto a metal substrate such as Cu, Ag, and Ta [60]. One of the reasons for the increased damping constant in this study can be due to spin injection to Si substrate [61], which causes an increase in the damping constant.

## 4. Conclusions

In this research, the AMR effect was achieved with Py thin films prepared by a simple and inexpensive method. These layers were applied on inexpensive Si substrates which gives the layers great potential for use in Si-based electronics. The electrodeposited Py thin films represented good adhesion and uniformity on the Si surface. AFM and FESEM results showed that the thickness and morphology of the layers grown by electrodeposition can be tuned and, in turn, the MOKE, AMR, and FMR response of the layers can be controlled. The effect of electrodeposition time (120 s and 150 s) on the thickness of Py thin films was investigated and different thicknesses of 56 and 70 nm were obtained, respectively. The results showed that the increase in the thickness results in higher roughness, coercivity, MOKE signal, and AMR. The damping constant for Py thin film with 56 nm thickness was calculated as 1.36 × 10^−2^, which is in the order of values achieved by sputtering. The proper magnetic characteristics along with the low-cost method and materials indicate that the growth method may be used for making industrial devices at a large scale with high functionality.
